# Chemotherapy induced neutropenia and febrile neutropenia among breast cancer patients in a tertiary hospital in Nigeria

**DOI:** 10.3332/ecancer.2021.1188

**Published:** 2021-02-15

**Authors:** Omolola Salako, Kehinde Sharafadeen Okunade, Adeoluwa Akeem Adeniji, Gabriel Timilehin Fagbenro, Oluwasegun Joshua Afolaranmi

**Affiliations:** 1 Radiodiagnosis, Radiation Biology and Radiotherapy Department, College of Medicine, University of Lagos, Lagos 100254, Nigeria; 2 Department of Obstetrics and Gynecology, College of Medicine, University of Lagos, Lagos 100254, Nigeria; 3 Oncology and Radiotherapy Department, Lagos University Teaching Hospital, Lagos 100254, Nigeria

**Keywords:** chemotherapy, CIN, febrile neutropenia, Nigeria

## Abstract

**Purpose:**

This study assessed the incidence of chemotherapy-induced neutropenia and febrile neutropenia (FN) while identifying their associated factors.

**Methods:**

A prospective cross-sectional study was conducted among 113 female chemotherapy-naïve breast cancer patients over a 2-year period. Socio-demographic, clinical and haematological data were obtained via semi-structured interviews and from medical case files. Blood samples for complete blood count parameters were collected 2 weeks after each course of chemotherapy. The National Cancer Institute Common Terminology Criteria for Adverse Events version 4.03 was used to assess FN, neutropenia and their severity.

**Results:**

The incidence of neutropenia and FN among the patients was 31.9% and 5.3%, respectively. Throughout all courses of chemotherapy (*n* = 502), there were 57 (11.4%) neutropenic episodes with 6.6% mild, 3.4% moderate and 1.4% severe neutropenia. The incidence of neutropenia decreased with increasing chemotherapy courses, with a rate of 14.2% and 4.9% after the first and last course, respectively. Factors associated with the risk of developing neutropenia include increasing age (*p* = 0.014), Eastern Cooperative Oncology Group performance score ≥ 1 at presentation (*p* = 0.033) and presence of bone metastasis (*p* = 0.002).

**Conclusion:**

One in three breast cancer patients in this study developed neutropenia while on chemotherapy but no independent risk factors were identified for FN among these patients. This study has, therefore, provided the preliminary data necessary for further independent validation of the identified risk factors for FN in a more robust and well-designed study within our clinical practice setting in Nigeria.

## Introduction

Neutropenia, in concert with its major complication, febrile neutropenia (FN), is a major dose-limiting adverse effect of systemic cancer chemotherapy. This relationship between chemotherapy and neutropenia referred to as chemotherapy-induced neutropenia (CIN) has been associated with significant morbidity and mortality, and huge costs of management in cancer patients [1]. CIN, defined as an absolute neutrophil count (ANC) less than 1,500 cell/μl occurring as a side effect of chemotherapeutic regimens, commonly results in FN (occurrence of fever with neutropenia) which requires inpatient evaluations and the use of empirical broad-spectrum antibiotics [1, 2]. The result of this is a reduction in chemotherapy doses and/or treatment delays which leads to worse clinical outcomes in the patients concerned [3, 4].

To counter the effects of CIN, granulocyte colony-stimulating factor (GCSF) was developed and this has been found to significantly lower the risk, severity and length of neutropenia and FN [5, 6]. According to the guidelines by the American Society of Clinical Oncology (ASCO), European Society for Medical Oncology, National Comprehensive Cancer Network and European Organisation for Research and Treatment of Cancer, it is important to evaluate both chemotherapy regimen risk and patient-specific risk factors when evaluating the risk of FN, and hence, the potential need for prophylactic G-CSF use [7–10]. ASCO guideline recommends primary prophylaxis with GCSF in patients who have an approximately 20% or higher risk for FN based on the patient’s characteristics, specific disease-condition and treatment-related factors while secondary prophylaxis is recommended for patients who experienced a neutropenic complication from a previous cycle of chemotherapy (for which primary prophylaxis was not received), in which a reduced dose or treatment delay may compromise disease-free or overall survival or treatment outcome [7].

However, despite the benefits of the use of G-CSF, there is largely a lack of adherence to the guidelines recommendations with several studies showing its underutilisation in patients undergoing chemotherapy treatments associated with a high-risk of FN, while being over-utilised in patients with a low-risk FN [11]. This inappropriate use of G-CSF is also evident among cancer patients on treatment in Nigeria and other parts of sub-Saharan Africa. As a result, this calls for a more appropriate use that requires identifying patients who are more at risk for neutropenic complications and directing the use of G-CSF at those who are most likely to benefit from its use as recommended by the standard guidelines [7–10, 12].

In developing countries such as Nigeria, there is a paucity of data on the magnitude of neutropenia and FN in cancer patients on chemotherapy. However, the experience of many practicing oncologists in Nigeria shows that these complications of chemotherapy occur very frequently, and as a result, the clinical outcomes in such patients usually become compromised. More so, because of the dearth of information about the nature of CIN and FN in Nigerian cancer patients, physicians usually do not look out for those who are more at risk to prevent their occurrence at the initiation of chemotherapy treatment. The dearth of information has also not helped to develop unifying treatment protocols to guide their management in such patients. This is why this study was aimed at generating information about the incidence of neutropenia and FN; and the factors associated with their occurrence in patients with breast cancer. The data generated through this study will help to better understand the magnitude and prevalence of CIN in breast cancer patients, considering the peculiarities of the Nigerian and African cancer patients and their response to chemotherapy. It will also help to determine the patient-specific-, disease- and treatment- factors that put some patients at greater risk of developing CIN and point at those who will benefit more from prophylactic use of G-CSF to ensure cost-effective applications. In addition, this study will evaluate the specific correlates of CIN, and assist in developing efficacious management protocols that will be effective in treating it in Nigerian and African cancer patients in general.

## Materials and methods

### Study design and setting

This is a prospective cross-sectional study among patients recruited from the Radiotherapy unit of Lagos University Teaching Hospital (LUTH), Idi-Araba, Nigeria. LUTH is the teaching hospital of the College of Medicine, University of Lagos. It acts mainly as a referral centre for other government-owned and private hospitals in the state. It is on the mainland of Lagos which has a population of over 17 million inhabitants. It has about 800-bed spaces.

### Study population and eligibility criteria

Participants selected were new histologically diagnosed, chemotherapy-naive breast cancer patients who attended the outpatient clinics for treatment for the first time from July 2017 to July 2019. Participants were all females aged 18 years or more. Patients who were acutely ill were excluded from the study.

### Data collection

A structured interviewer-administered proforma was used to obtain the required data from all study participants during the study period. The proforma collected data on socio-demographics, disease and treatment characteristics; and chemotherapy-induced complications. Venous blood sample was collected after 2 weeks of every chemotherapy session and its cell parameters were analysed and recorded in preparation for the next session. Neutropenia and FN were determined and graded using the Common Terminology Criteria for Adverse Events version 4.03 [13]. Reduction in ANC was graded as mild, moderate or severe. Mild neutropenia is present when the ANC is 1,000–1,500 cells/μL, moderate neutropenia is present with an ANC of 500–1,000/μL and severe neutropenia refers to an ANC lower than 500 cells/μL. FN is characterised by any grade of reduced ANC and a single body temperature measurement of 38.3°C (101°F) or a sustained temperature of >38°C (100.4°F) for more than 1 hour.

### Statistical analysis

Data analysis was done using Statistical Package for Social Sciences software for Windows (version 21; SPSS, Chicago, IL). Socio-demographic, risk factors and clinical data of respondents, as well as the tumour biology and immunohistochemistry, were analysed using descriptive statistics and presented in the form of frequencies, percentages, means and standard deviation. Normality of numerical variables was determined using the Shapiro–Wilk test. Categorical data were analysed as a percentage using the Chi-square test. The means of normally distributed variables were compared using the *t*-test while those not normally distributed were summarised as medians (interquartile range) and compared using the Mann–Whitney U test. Statistical significance was set at ≤0.05.

### Ethical considerations

The study was conducted following ethical guidelines approval of the Institutional Review Board of LUTH, and all included study participants gave voluntary and informed consent. Ethical principles according to Helsinki’s declaration were observed throughout the conduct of the study.

## Results

A total of 113 patients, with histological-diagnosed breast cancer who are chemotherapy-naive, were recruited for this study. Their mean age was 49.5 years with a range of 19–75 years. The majority (84.1%) lives with a partner and 17.9% are unemployed (Table 1).

Most of them (78.8%) presented to the clinic within 6 months of the onset of symptoms. Only 1.8% had an Eastern Cooperative Oncology Group (ECOG) performance score greater than 1. The mean body mass index (BMI) at the first presentation to the clinic was 28.2 kg/m^2^, with 69.0% (78/113) of the patients being overweight and/or obese. Thirty-six patients (27.0%) have had breast surgery done including mastectomy in 16.0% and lumpectomy in 11.0%. Up to 28.9% had comorbidities, with hypertension (21.0%) and diabetes (5.3%) being the most common types (Table 2).

The tumour site distribution for the left, right and bilateral sides are 43.4%, 52.2% and 4.4%, respectively. The classification of the patients based on cancer staging shows that 4 (3.5%) were in stage 1, 7 (6.2%) were in stage 2, 81 (71.7%) were in stage 3 and 21 (18.6%) were in stage 4. Tumour classification was 15.0% (Grade I), 59.3% (Grade II) and 19.5% (Grade III). Invasive ductal type (89.4%) was the most common histology type (Table 3).

Table 3 also summarises the immunohistochemical status available for 86 patients. Estrogen receptor (ER) and progesterone receptor (PR) positivities were 36 cases (41.9%) and 22 (25.6%), respectively. About one in every five (17.4%) participants had HER-2 positive receptor status.

Neoadjuvant treatment plan (69.9%) was the most commonly adopted. Anthracycline-based chemotherapy combination was most commonly employed at the start of treatment of the patients and this was used in 98.2% of cases. In subsequent or second-line chemotherapy courses, taxane-based combinations (80.6%) were the most commonly introduced medication. Most (*n* = 111, 98.2%) of the participants were started on an anthracycline-based regimen, out of which 40 were later switched to a second regimen that comprises 97.2% (39/40) taxane-based therapy (Table 4).

**Figure 1.**
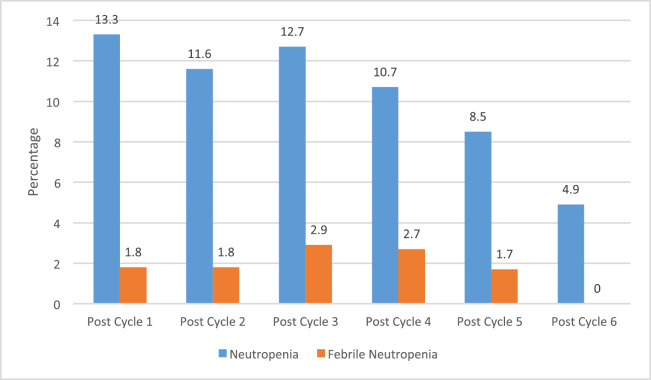
Incidence of neutropenia and FN among the patients.

A total of 502 chemotherapy sessions were recorded among the 113 patients. The incidence of neutropenia and FN according to the total chemotherapy session is 11.4% (57/502) and 2.2% (11/502), respectively. Also, 31.9% (36/113) and 5.3% (6/113) of the patients had at least one episode of neutropenia and FN, respectively, during chemotherapy (Figure 1).

**Figure 2.**
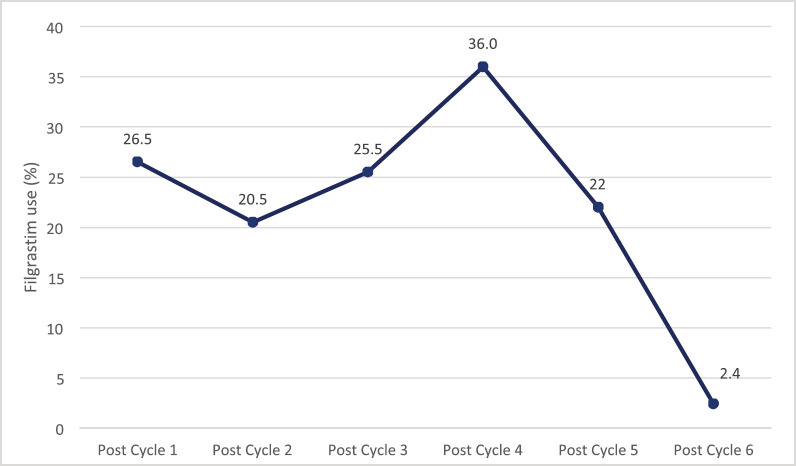
Trend of G-CSF (Filgrastim) use among patients during chemotherapy.

The trend of use of G-CSF increases progressively until a peak rate of 36.0% after the fourth cycle of chemotherapy following which its utilisation decreases progressively to 2.4% after the sixth cycle (Figure 2).

The identified factors associated with neutropenia among patients in this study include older age (≥50 years), ECOG score greater than 1 and the presence of bone metastasis (Table 6). None of the factors was significantly associated with FN.

## Discussion

In this prospective, observational study of 113 chemotherapy-naïve breast cancer patients, the mean age of 49.5 years, as well as other socio-demographic, clinical and histopathological characteristics is mostly in keeping with previous reports on breast cancer epidemiology in Nigeria [14, 15]. However, the lower mean age of the participants in this study compared to other studies from Europe and USA [16, 17] further suggests that the Nigeria breast cancer patients are of younger age group compared to their counterparts in Europe and the USA due to the relatively lower life-expectancy in our part of the world. The larger proportion of patients that present with an advanced stage disease may also be attributed largely to the high level of illiteracy, ignorance and lack of access to the basic screening and diagnostic facilities such as mammography among the majority of the Nigerian female population. Majority of the women in this study are either overweight or obese as expected with obesity being a recognised modifiable risk factor for breast cancer [18]. This finding could also have a significant impact on the risk and incidence of CIN and FN due to reduction in the dose of chemotherapy agents as a result of dose calculations based mainly on the use of a maximum bode surface area of 2.2 square meter as generally practised in our setting.

Altogether, this study captured a total of 502 chemotherapy sessions, of which there were 67 neutropenic and 11 febrile neutropenic episodes recorded. This neutropenic effect of cancer chemotherapy is well documented in the literature [1, 19, 20]. In a Nigerian study that measured ANC of cancer patients, half of which were breast cancer patients, before and during chemotherapy, a nearly 33% drop in mean ANC was observed by day 12 on chemotherapy [21].

Almost a third (31.9%) of the patients had at least one episode of neutropenia while only 5.3% had at least an episode of FN. Although studies documenting the incidence of CIN and FN in this region are scarce, very similar results have emanated from large and robustly designed studies. The PRAXIS prospective multicentre study observed a 4.3% incidence of FN among 734 patients [22] and a European multinational neutropenia study that included 444 breast cancer patients reported an FN incidence of 6% [23]. Other recent single centre studies have even reported much higher values, with one Brazilian group reporting as high as a 63.3% [24] incidence of CIN (*N* = 79), a study from France with 524 breast cancer patients reported a 17% incidence of FN [25] and a UK study of 325 patients showing a 19% incidence of FN [26].

The risk of developing neutropenia during chemotherapy increased significantly with age, ECOG performance score of >1 and bone metastasis. Baghlaf *et al* [27], in their study of breast cancer patients in Saudi Arabia, identified age, non-anthracycline or taxane-based regimen and neo-adjuvant chemotherapy as having statistically significant associations with the development of FN. However, our study did not show a significant relationship between taxane-based regimen and CIN or FN risk, which may be attributable to the fact that most (98.2%) of the participants started on an anthracycline-based regimen, out of which 40 patients were later switched to a taxane-based second regimen for various reasons ranging from intolerable side effects to failure of response to the first-line chemotherapy regimen. Performance status has also been shown to be an independent predictor of CIN and FN [28], as documented in this study.

Notably, bone metastasis was also associated with increased risk of neutropenic episodes in this study. The bone is a very frequent site of breast cancer metastasis, with over 60% bone involvement observed in patients with metastatic breast cancer at initial presentation [29]. The osteolytic and osteoblastic effects of bone metastasis can lead to bone marrow aplasia, contributing to the risk of CIN and FN [30]. Other previously reported risk factors include lower BMI, higher doses, lower baseline white cell count, advanced disease, comorbidities and genetic factors [31–33].

There is overwhelming evidence on the efficacy of GCSF as prophylaxis for CIN and FN [25, 34, 35]. In a retrospective cohort study done in the United States, Weycker *et al* [34] observed that almost half of all FN hospitalisations occurred in patients who did not receive GCSF prophylaxis in that cycle compared to only 8.8% among those who received. With serious limitations in access to newer kinds of therapies coupled with the peculiarities in prevalent subtypes and drug response of breast cancer in Africa [36], cytotoxic chemotherapy combined with surgery is still the mainstay of treatment. In spite of this, the use of GCSF is still very scarce in Nigeria and indeed in most other developing countries, leading to an aggravated impact of CIN and FN occurrence on the cost of care, disruption of treatment, dose reduction and clinical outcome of breast cancer.

As shown in this study, the number of patients decreases progressively from the first course to the sixth course of chemotherapy treatment and this could be attributed mostly to the intolerable side effects and cost of the drugs and the treatment fatigue commonly found among patients with chronic disease conditions including cancer in this part of the world. However, the incidence of CIN or FN is highest after the first course of chemotherapy, with a decreasing trend as the number of courses increases. This pattern corroborated the available evidence in the literature [25, 27] and this may be attributed to the introduction of GCSF in subsequent cycles of chemotherapy. Hence, the use of GCSF should not only be commenced after the development of CIN or FN but on the initiation of chemotherapy and subsequently based on the patient’s risk category. Indeed, the maximum benefit is derived from GCSF use in CIN/FN prophylaxis when strict adherence to standard guidelines is observed [9, 34].

We noted a few limitations in this study and these include the small sample size, unavailability of data on the impact of chemotherapy dose adjustments, discontinuation and switching of treatment, clinical outcomes and introduction of GCSF on the likelihood of underestimation of the diagnosis and incidence of CIN or FN, and may also introduce bias in concluding the different risk of CIN/FN. Finally, we did not make adjustments for multiple testing in the statistical analysis due to the relatively small sample size used and also the few recorded cases of FN could be the reason for the absence of any significant association with any of the tested factors in the study. However, findings from this study provide an initial insight into how common these complications are in our environment and provide preliminary evidence to guide routine clinical use of prophylactic G-CSF at the initiation of breast cancer chemotherapy, particularly in at-risk groups as identified in this study and others.

## Conclusion

One in three breast cancer patients in this study developed neutropenia while on chemotherapy but no independent risk factors were identified for FN among these patients. This suggests that the use of prophylactic G-CSF could be appropriate for a predefined subgroup of breast cancer patients with an increased risk of neutropenia such as the elderly, unstable patients and those with bone metastasis as observed in this current study. This study has, therefore, provided the preliminary data necessary for further independent validation of these identified risk factors in a more robust and well-designed study within our clinical practice setting in Nigeria. In the meantime, a great effort should be instituted to educating patients as much as possible and securing a quick and effective treatment with G-CSF whenever necessary.

## Conflicts of interest

None.

## Funding

This work was supported by a Memorial Sloan Kettering Cancer Centre Global Cancer Disparities Pilot Grant.

## Figures and Tables

**Table 1.** Sociodemographic characteristics of study participants.

Variable (*n* = 113)	Frequency (%)
**Age (years)** Young (<44) Middle age (45–65) Elderly (>65) **Mean age: 49.5 ± 11.8**	48 (42.5) 57 (50.4) 8 (7.1)
**Occupation** Professional Skilled Semi-skilled Unskilled Unemployed	26 (23.0) 16 (14.2) 41 (36.3) 18 (15.9) 12 (10.6)
**Marital status** Living with a partner Not living with a partner^a^	95 (84.1) 18 (15.9)
**Religion** Christian Muslim	96 (85.0) 17 (15.0)
**Tribe** Igbo Yoruba Others (Hausa, Calabar, Delta)	40 (35.4) 54 (47.8) 19 (16.8)

a Divorced, single, widow

**Table 2.** History of hospital presentation and clinical information of study participants.

Variable (*n* = 113)	Frequency (%)
**The duration between the onset of symptoms and first presentation to LUTH ** ≤6 months >6 months	89 (78.8) 24 (21.2)
**ECOG performance score at presentation** 0 1 2	67 (59.3) 44 (38.9) 2 (1.8)
**Nutritional status** Underweight Normal Overweight/obese	2 (1.8) 33 (29.2) 78 (69.0)
**Breast surgery history** Lumpectomy Mastectomy None	12 (11.0) 18 (16.0) 83 (73.0)
**Comorbidity** AIDS Diabetes Hypertension Hyperthyroidism Peptic ulcer disease None	2 (1.8) 5 (5.3) 23 (21.0) 2 (1.8) 3 (2.7) 78 (71.1)

**Table 3.** Pathological information of study participants.

Variable (*n* = 113)	Frequency (%)
**Primary site of disease** Left Right Bilateral	49 (43.4) 59 (52.2) 5 (4.4)
**Bilateral type (*n* = 5)** Metachronous Synchronous	3 (60.0) 2 (40.0)
**Histological type** Invasive ductal carcinoma Invasive lobular carcinoma Others (Medullary, mucinous, papillary, metastatic)	101 (89.4) 6 (5.3) 6 (5.3)
**Grade of breast cancer (*n* = 106)** I II III	17 (16.0) 67 (63.2) 22 (20.8)
**Tumour size** T1 T2 T3 T4	4 (3.6) 15 (13.3) 45 (39.8) 49 (43.4)
**Nodal status** N0 N1 N2 N3	9 (8.0) 52 (46.0) 49 (43.4) 3 (2.7)
**Stage of disease** I II III IV	4 (3.5) 7 (6.2) 81 (71.7) 21 (18.6)
**Metastatic site^a^ (*n* = 21)** Bone Liver Lungs	2 (9.5) 5 (23.8) 18 (85.7)
**Immunohistochemistry** ER+ PR+ HER-2+ HER-2 equivocal Triple negative	36 (41.9) 22 (25.6) 15 (17.4) 4 (4.7) 40 (46.5)

a Multiple options allowed

**Table 4.** Treatment plan of study participants.

Variable	Frequency
**Chemotherapy indication** Adjuvant Neoadjuvant Palliative	25 (22.1) 79 (69.9) 9 (8.0)
**Chemotherapy (first-line regimen)** Anthracycline Cyclophosphamide, Methotrexate, Fluorouracil Taxane	111 (98.2) 1 (0.9) 1 (0.9)
**Chemotherapy (second-line regimen) (*n* = 40)** Platinum Taxane Platinum and taxane	1 (2.4) 33 (80.6) 6 (14.6)

**Table 5.** Severity of neutropenia among patients.

Chemotherapy cycle	Neutropenia (ANC) *n* (%)			Total *n* (%)
	Mild (>1,000–1,500 cells/μL)	Moderate (500–1,000 cells/μL)	Severe (<500 cells/μL)	
Post cycle 1 (*n* = 113)	9 (8.0)	6 (5.3)	1 (0.9)	16 (13.3)
Post cycle 2 (*n* = 112)	10 (8.9)	3 (2.7)	0 (0.0)	13 (11.6)
Post cycle 3 (*n* = 102)	5 (4.9)	6 (5.9)	2 (1.9)	13 (12.7)
Post cycle 4 (*n* = 75)	3 (4.0)	2 (2.7)	3 (4.0)	8 (10.7)
Post cycle 5 (*n* = 59)	4 (6.8)	0 (0.0)	1 (1.7)	5 (8.5)
Post cycle 6 (*n* = 41)	2 (4.9)	0 (0.0)	0 (0.0)	2 (4.9)
Total (*n* = 502)	33 (6.6)	17 (3.4)	7 (1.4)	57 (11.4)

**Table 6.** Factors associated with neutropenia and FN among patients. p-values less than 0.005 are statistically significant.

Variables	Neutropenia		FN	
	Odds ratio	*p*-value	Odds ratio	*p*-value
Age (≥50years) Presence of comorbidity Late presentation to hospital ECOG score ≥ 1 Overweight/obesity Advanced stage Presence of bone metastasis Taxane-based regimen	1.098 3.925 0.984 1.190 4.812 0.847 3.094 7.352	**0 .014** 0.172 0.429 **0.033** 0.055 0.477 **0.002** 0.314	2.614 3.713 1.184 0.312 5.211 7.612 2.588 1.103	0.984 0.362 0.218 0.482 0.662 0.978 0.872 0.418
